# Unexpected Diagnosis of Uterine Adenosarcoma With Sarcomatous Overgrowth After Hysteroscopic Polypectomy for a Presumed Endometrial Polyp

**DOI:** 10.7759/cureus.114602

**Published:** 2026-08-16

**Authors:** Nobuhiko Suzuki, Yuko Horiuchi, Naoyuki Iwahashi, Yurina Mikasa, Kazuhiko Ino

**Affiliations:** 1 Department of Obstetrics and Gynecology, Wakayama Medical University, Wakayama, JPN; 2 Department of Human Pathology, Wakayama Medical University, Wakayama, JPN

**Keywords:** gynecology, hysteroscopic polypectomy, oncology, sarcomatous overgrowth, uterine adenosarcoma

## Abstract

Uterine adenosarcoma is a rare biphasic malignancy composed of benign epithelial and malignant stromal components. Adenosarcoma with sarcomatous overgrowth is an aggressive subtype associated with rapid progression and a poor prognosis. We report a case of uterine adenosarcoma with sarcomatous overgrowth diagnosed through the histopathological evaluation of a hysteroscopic polypectomy specimen. A 47-year-old woman presented to a local physician with abnormal uterine bleeding. Pelvic magnetic resonance imaging revealed a 5.5-cm polypoid lesion arising from the uterine corpus and protruding into the endometrial cavity, along with an intramyometrial mass. Hysteroscopic polypectomy was performed, and histopathological examination revealed uterine adenosarcoma, prompting referral to our institution. Positron emission tomography/computed tomography demonstrated fluorodeoxyglucose uptake in both the endometrial lesion and intramyometrial mass, without evidence of distant metastasis. The patient underwent total abdominal hysterectomy with bilateral salpingo-oophorectomy. Histopathological examination of the resected uterus showed mildly atypical glandular epithelium surrounded by densely cellular, high-grade malignant stromal cells, together with extensive high-grade sarcomatous overgrowth involving the myometrium. Immunohistochemical staining demonstrated strong CD10 positivity and focal weak h-caldesmon positivity. The final diagnosis was uterine adenosarcoma with sarcomatous overgrowth, International Federation of Gynecology and Obstetrics stage IC. The patient received three cycles of adjuvant doxorubicin plus ifosfamide chemotherapy and remained free of recurrence 18 months after surgery. This case highlights the diagnostic value of hysteroscopic polypectomy in patients with intracavitary uterine lesions and underscores the importance of careful histopathological evaluation for the early recognition of sarcomatous overgrowth.

## Introduction

Uterine adenosarcoma is a rare biphasic malignancy composed of benign epithelial and malignant stromal components. Although conventional uterine adenosarcoma is generally considered a low-grade malignancy, adenosarcoma with sarcomatous overgrowth represents an aggressive subtype associated with rapid progression, recurrence, and poor prognosis [1,2]. Because its clinical and imaging findings may mimic benign endometrial polyps or submucosal lesions, preoperative diagnosis can be challenging. We report a case of uterine adenosarcoma with sarcomatous overgrowth diagnosed through histopathological evaluation of a hysteroscopic polypectomy specimen.

The preoperative diagnosis of uterine adenosarcoma based on imaging findings alone is challenging. Imaging modalities play an important role in the evaluation of uterine intracavitary pathologies and malignant pelvic tumors. Transvaginal B-mode ultrasound is widely used as a first-line imaging tool, and Doppler ultrasound can provide additional information regarding vascularity and lesion characterization in women with perimenopausal and postmenopausal bleeding [3]. In addition, imaging modalities such as ultrasound, CT, and MRI are useful for assessing tumor location, morphology, vascularity, local extension, and differential diagnosis in suspected malignant pelvic tumors, including uterine sarcoma [4]. Therefore, appropriate imaging assessment is essential for clinical decision-making and surgical planning in patients with pelvic tumors. These tumors frequently present as polypoid masses protruding into the uterine cavity and may be difficult to distinguish from benign endometrial polyps. We herein report a case that was initially suspected to be an endometrial polyp but was diagnosed as uterine adenosarcoma based on the histopathological examination of a hysteroscopic polypectomy specimen. Following hysterectomy, the final diagnosis was uterine adenosarcoma with sarcomatous overgrowth. The patient subsequently received adjuvant chemotherapy and has remained free of recurrence.

## Case presentation

**Figure 1:**
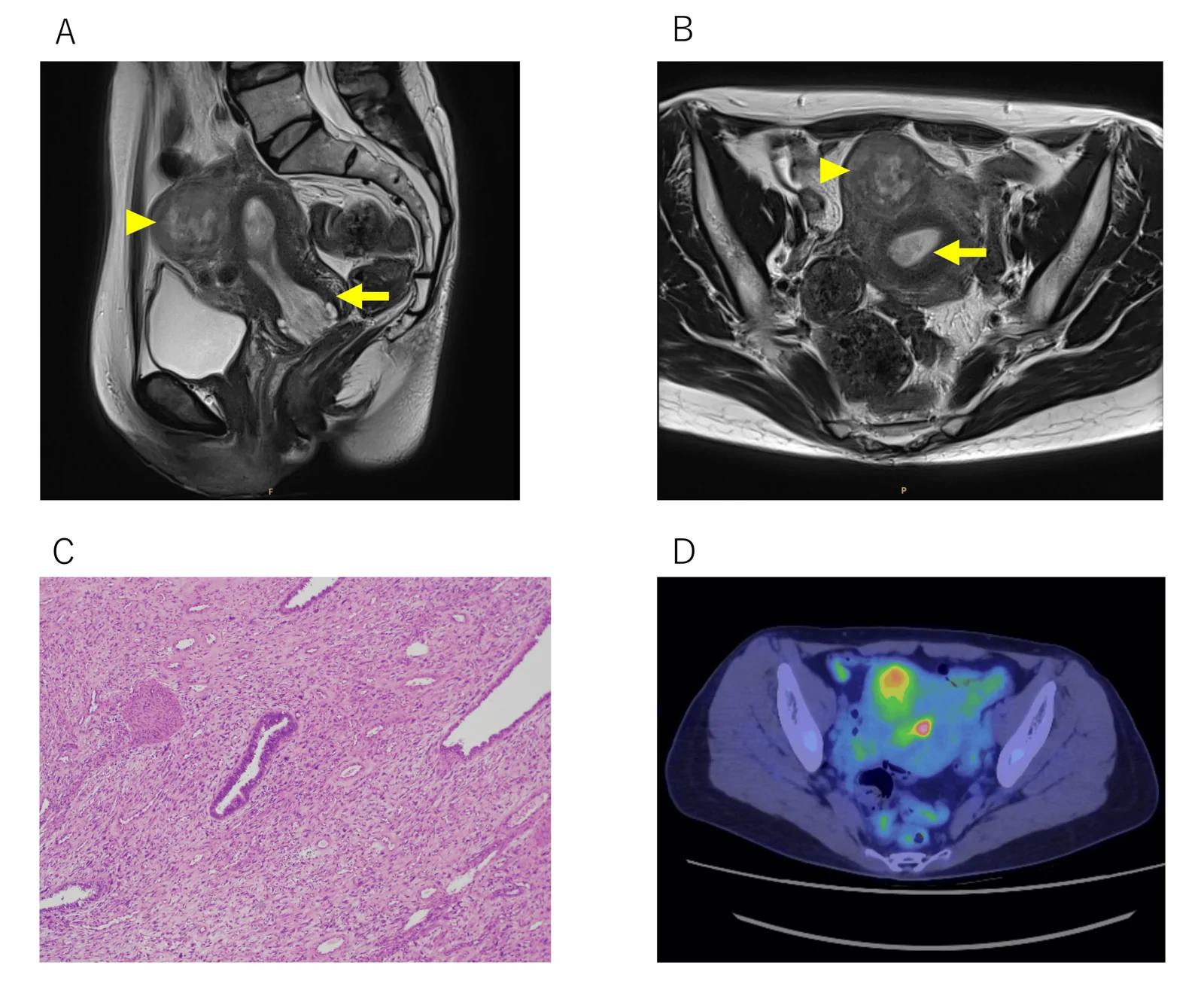
Preoperative Imaging and Histopathological Findings of the Uterine Polypoid and Intramyometrial Lesions A and B: Pelvic magnetic resonance imaging on T2-weighted images showing a polypoid lesion in the uterine cavity (arrow) and a mass in the anterior myometrium (arrowhead). C: Histopathological findings of the hysteroscopically resected polypoid lesion with hematoxylin and eosin staining (magnification, ×40). D: Positron emission tomography/computed tomography showing fluorodeoxyglucose uptake in the endometrial and intramyometrial lesions.

A 47-year-old woman, gravida 2, para 2, presented to her previous physician with irregular menstruation and abnormal uterine bleeding. Her medical history was significant for cervical conization performed for cervical intraepithelial neoplasia grade 2. Her family history was unremarkable. Pelvic magnetic resonance imaging (MRI) performed at the referring hospital revealed a 5.5-cm polypoid lesion arising from the uterine corpus and protruding into the uterine cavity, extending to the external cervical os. The lesion showed predominantly high signal intensity on T2-weighted images, with scattered low-signal-intensity cystic areas. In addition, an intramyometrial mass in the anterior wall of the uterine corpus, suspected to represent a degenerated leiomyoma, was identified (Figure 1A, 1B). No abnormalities were detected in either adnexa. Laboratory investigations, including serum tumor marker levels, were within normal limits, and cervical cytology was negative. 

The patient underwent hysteroscopic polypectomy at the referring hospital under a preoperative diagnosis of an endometrial polyp. Histopathological examination of the resected polyp demonstrated irregularly dilated and branching endometrial glands with minimal cytologic atypia, some of which exhibited a phyllodiform, cleft-like architecture. The stroma was fibrous, with increased cellularity surrounding the glands (Figure 1C). The stromal spindle cells had enlarged, pleomorphic nuclei that varied markedly in size. Immunohistochemical staining showed that the atypical stromal cells were positive for estrogen receptor (ER) and CD10 and negative for α-smooth muscle actin. Based on these findings, the resected polyp was diagnosed as uterine adenosarcoma, and the patient was referred to our institution for further management.

Positron emission tomography/computed tomography (PET/CT) demonstrated fluorodeoxyglucose (FDG) uptake in the endometrium (SUVmax, 10.50) and the intramyometrial mass (SUVmax, 5.00) (Figure 1D). No findings suggestive of lymph node or distant metastasis were identified. Residual uterine adenosarcoma was suspected, and the intramyometrial lesion was considered to represent either myometrial invasion by the adenosarcoma or a coexisting degenerated leiomyoma. Therefore, total abdominal hysterectomy with bilateral salpingo-oophorectomy was performed. No peritoneal dissemination was observed intraoperatively, and peritoneal cytology was negative.

**Figure 2:**
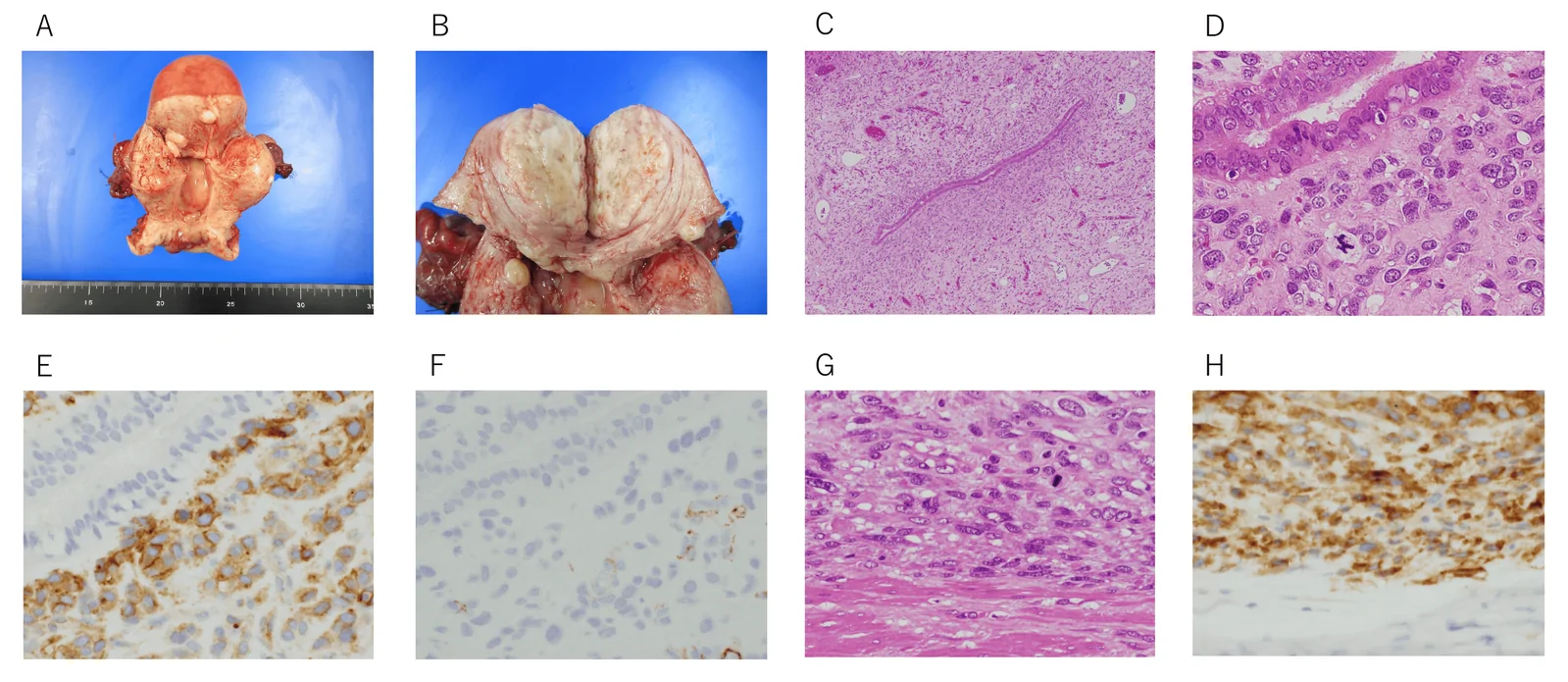
Gross and Histopathological Findings of the Resected Uterus A and B: Gross appearance. C-F: HE staining and immunohistochemical staining of the residual polyp-like lesions. C and D, HE staining, E: CD10, F: h-Caldesmon. G and H: HE staining and immunohistochemical staining of the myometrium mass. G: HE staining, H: CD10. Magnification: C, ×40; D-H, ×400.

Gross examination of the resected uterus revealed a residual polypoid tumor, while the intramyometrial lesion appeared whitish and contained focal areas of hemorrhage (Figure 2A, 2B). Histopathological examination of the residual polypoid lesion showed sparsely distributed glands with preserved architecture and minimal cytologic atypia. In contrast, marked stromal expansion with diffuse and fascicular proliferation of atypical spindle cells was observed. Mildly atypical glandular epithelium formed glands surrounded by densely cellular atypical stromal cells, demonstrating the characteristic pattern of periglandular cuffing (condensation) (Figure 2C, 2D). The stromal cells exhibited nuclear enlargement, hyperchromasia, irregular nuclear contours, and scattered mitotic figures. Immunohistochemically, the stromal cells showed diffuse positivity for CD10 and progesterone receptor (PgR), whereas h-caldesmon and desmin were only focally and weakly positive (Figure 2E, 2F).

Histopathological examination of the intramyometrial lesion similarly demonstrated extensive proliferation of highly atypical spindle cells arranged in fascicles, indicating myometrial involvement by the sarcomatous overgrowth component. Although precise measurement of the invasion depth was difficult because of the polypoid lesion and intramyometrial tumor component, the tumor extended beyond one-half of the myometrial thickness. There was no cervical stromal involvement, serosal involvement, or extrauterine disease. The immunohistochemical findings were similar to those of the polypoid lesion, including CD10 positivity. The MIB-1 labeling index reached 60% in the hotspot area, and the lesion was diagnosed as adenosarcoma with sarcomatoid changes (Figure 2G, 2H). Based on these findings, the final diagnosis was uterine adenosarcoma with sarcomatous overgrowth, FIGO (International Federation of Gynecology and Obstetrics) stage IC.

After surgery, the patient received three cycles of doxorubicin plus ifosfamide (AI therapy) at four-week intervals. She has remained free of recurrence for 18 months since completing treatment.

## Discussion

Uterine adenosarcoma is a rare malignancy accounting for approximately 5% of uterine sarcomas. Histologically, it is a biphasic tumor composed of benign glandular epithelium and malignant stromal components [1]. The median age at diagnosis is 54 years, although approximately 30% of patients are premenopausal [2,5]. Conventional adenosarcoma generally has a more favorable prognosis than other uterine sarcomas. According to the current WHO Classification of Tumours, adenosarcoma with sarcomatous overgrowth is defined by the presence of a pure sarcomatous component lacking benign epithelial elements that occupies at least 25% of the tumor [6]. This definition is consistent with earlier reports by Clement and Scully and subsequent clinicopathological studies [2,7]. This subtype is associated with aggressive behavior and a markedly higher recurrence rate. Reported recurrence rates are 26-40% for adenosarcoma overall and 70-82% for tumors with sarcomatous overgrowth [1,2,5,7,8]. No adjuvant treatment has yet been conclusively shown to improve outcomes in these patients. The present case is notable because uterine adenosarcoma was diagnosed from a hysteroscopically resected polyp, while sarcomatous overgrowth was identified only after hysterectomy.

Abnormal uterine bleeding is the most common presenting symptom of uterine adenosarcoma, although pelvic pain, vaginal discharge, and uterine polyps have also been reported [1,7,8]. On imaging, these tumors often appear as polypoid masses protruding into the uterine cavity and may resemble benign endometrial polyps or other intracavitary lesions. In the present case, hysteroscopic polypectomy was initially performed for a presumed endometrial polyp, and the diagnosis of adenosarcoma was established histologically. Characteristic magnetic resonance imaging findings include heterogeneous high signal intensity on T2-weighted images with intermixed cystic areas and areas of high signal intensity on T1-weighted images caused by hemorrhage [9,10]. These features were present in the polypoid lesion. Adenosarcoma with sarcomatous overgrowth may also contain irregular nonenhancing areas reflecting central necrosis [9]. Although definite necrosis was not identified in the intramyometrial lesion in this case, its heterogeneous T2-weighted signal and fluorodeoxyglucose uptake on positron emission tomography/computed tomography were retrospectively considered compatible with myometrial involvement by the sarcomatous overgrowth component.

Histopathological examination of the hysteroscopically resected polyp established the diagnosis of adenosarcoma, but sarcomatous overgrowth was not evident in the limited specimen. In contrast, the hysterectomy specimen showed extensive proliferation of highly atypical spindle cells, with the high-grade sarcomatous component occupying more than 25% of the tumor. The MIB-1 labeling index reached 60%, supporting the diagnosis of adenosarcoma with sarcomatous overgrowth. Immunohistochemically, the tumor showed diffuse CD10 positivity and only focal h-caldesmon positivity, consistent with the immunophenotype of adenosarcoma. Although loss or reduced expression of estrogen and progesterone receptors has been reported in sarcomatous overgrowth [11], progesterone receptor expression was retained in the present case.

The standard surgical treatment for uterine adenosarcoma is total hysterectomy with bilateral salpingo-oophorectomy. In the surgical management of uterine tumors of uncertain malignant potential, avoidance of tumor fragmentation is also important. Although parasitic tumor formation has been reported mainly after laparoscopic power morcellation for uterine fibroids, this risk underscores the importance of careful preoperative assessment and en bloc tumor removal when uterine sarcoma is suspected [12]. Because lymph node metastasis is uncommon, routine lymphadenectomy is generally not recommended [2,13,14]. Accordingly, the patient underwent total abdominal hysterectomy with bilateral salpingo-oophorectomy without lymphadenectomy. Adverse prognostic factors include advanced stage, deep myometrial invasion, high tumor grade, a high mitotic index, heterologous sarcomatous elements, and sarcomatous overgrowth [2,14]. Owing to the rarity of the disease, the role of adjuvant therapy remains uncertain. Although patients with stage I adenosarcoma with sarcomatous overgrowth are more likely to receive adjuvant treatment, a significant improvement in time to recurrence has not been demonstrated [15]. Nevertheless, doxorubicin plus ifosfamide may be considered in patients at high risk of recurrence [14]. In a study of recurrent uterine adenosarcoma by Nathenson et al., 28 of 59 patients who received first-line systemic therapy were treated with doxorubicin-containing regimens, including nine who received doxorubicin plus ifosfamide [1]. Given the high recurrence risk associated with sarcomatous overgrowth, adjuvant doxorubicin plus ifosfamide was administered in the present case. The patient has remained free of recurrence for 18 months; however, longer follow-up is required.

This case highlights the diagnostic challenge of uterine adenosarcoma with sarcomatous overgrowth presenting as an apparent endometrial polyp. Careful histopathological evaluation of hysteroscopically resected lesions, combined with appropriate imaging assessment and complete evaluation of the hysterectomy specimen, is important for identifying aggressive components that may not be evident in a limited specimen.

## Conclusions

In this case, uterine adenosarcoma was diagnosed through histopathological examination of a hysteroscopically resected intrauterine polypoid lesion, and subsequent examination of the hysterectomy specimen established the diagnosis of adenosarcoma with sarcomatous overgrowth. Given the aggressive behavior and poor prognosis associated with sarcomatous overgrowth, the patient received postoperative doxorubicin plus ifosfamide chemotherapy and has remained free of recurrence for 18 months. Although rare, uterine adenosarcoma should be considered in the differential diagnosis of large polypoid intrauterine lesions presenting with abnormal uterine bleeding.
